# Osteopontin deletion attenuates cyst growth but exacerbates fibrosis in mice with cystic kidney disease

**DOI:** 10.14814/phy2.70038

**Published:** 2024-09-05

**Authors:** Kyle P. Jansson, Jordan Kuluva, Shiqin Zhang, Taylor Swanson, Yan Zhang, Kurt A. Zimmerman, Timothy A. Fields, Darren P. Wallace, Peter S. Rowe, Jason R. Stubbs

**Affiliations:** ^1^ The Jared Grantham Kidney Institute University of Kansas Medical Center Kansas City Kansas USA; ^2^ Division of Nephrology and Hypertension, Department of Internal Medicine University of Kansas Medical Center Kansas City Kansas USA; ^3^ Division of Nephrology, Department of Internal Medicine University of Oklahoma Health Sciences Center Oklahoma City Oklahoma USA; ^4^ Pathology and Laboratory Medicine University of Kansas Medical Center Kansas City Kansas USA

**Keywords:** fibrosis, matricellular proteins, mineral metabolism, Osteopontin, PKD

## Abstract

Osteopontin (OPN) is a multi‐functional glycoprotein that coordinates the innate immune response, prevents nanocrystal formation in renal tubule fluid, and is a biomarker for kidney injury. OPN expression is markedly increased in cystic epithelial cells of polycystic kidney disease (PKD) kidneys; however, its role in PKD progression remains unclear. We investigated the in vitro effects of recombinant OPN on the proliferation of tubular epithelial cells from PKD and normal human kidneys and in vivo effects of OPN deletion on kidney cyst formation, fibrosis, and mineral metabolism in *pcy/pcy* mice, a non‐orthologous model of autosomal‐dominant PKD. In vitro studies revealed that OPN enhanced the proliferation of PKD cells but had no effect on normal kidney cells. Deletion of OPN in *pcy/pcy* mice significantly reduced kidney cyst burden; however, this was accompanied by increased fibrosis and no change in kidney function. The loss of OPN had no effect on kidney macrophage numbers, cyst epithelial cell proliferation, or apoptosis. Furthermore, there was no difference in kidney mineral deposition or mineral metabolism parameters between *pcy/pcy* mice with and without OPN expression. Global deletion of OPN reduced kidney cyst burden, while paradoxically exacerbating kidney fibrosis in mice with cystic kidney disease.

## 1 INTRODUCTION

Aberrant epithelial cell proliferation, macrophage infiltration, and tubulointerstitial fibrosis are important contributors to the progression of autosomal dominant polycystic kidney disease (PKD) (Karihaloo et al., 2011; Lanoix et al., 1996; Nadasdy et al., 1995; Norman, 2011; Raman et al., 2017; Ramasubbu et al., 1998; Swenson‐Fields et al., 2013; Ta et al., 2013; Yang et al., 2018); however, factors promoting these findings remain incompletely characterized. Recent evidence suggests that phosphate loading and tubular crystal deposition may serve an important role in promoting these events and associated cyst formation (Omede et al., 2020; Torres et al., 2019). Thus, it is plausible that pathways involved in mineral homeostasis may be key regulators of PKD progression.

Osteopontin (OPN), encoded by the secreted phosphoprotein 1 (*Spp1*) gene, is a multi‐functional protein that is produced by tubular epithelial cells and is present at high concentrations in urine (Xie et al., 2001). The name “osteopontin” is derived from the Latin words for “bone” and “bridge”, since it functions to facilitate the attachment of mineral aggregates to extracellular matrix or cells. Accordingly, OPN contains a phosphorylated poly‐aspartate region (ASARM motif) that tightly binds calcium‐phosphate complexes (Hoyer et al., 2001; Sorensen et al., 1995), and a separate integrin‐binding region (RGD sequence) that is a potent signal for leukocyte recruitment and fibrosis (Scatena et al., 2007; Xie et al., 2001). OPN is a potent enhancer of mineral solubility in urine where mineral concentrations often exceed supersaturation (Schlieper et al., 2007). As such, our group recently demonstrated that OPN deficiency in mice with decrements in kidney function resulted in a severe nephrocalcinosis phenotype (Stubbs et al., 2022). Moreover, we and others have shown that OPN expression is substantially increased in tubular epithelial cells in rodents with cystic kidney disease (Cowley Jr. et al., 2001; Stubbs et al., 2022).

Since tubular fluid phosphate is markedly elevated with nephron loss (Bank N et al., 1978), we theorized that increased tubular OPN production is an adaptive response to prevent crystal aggregation in supersaturated tubular fluid. However, since OPN has additional functions as both a promoter of cellular division and mediator of innate immune responses (Cui et al., 2007; Denhardt et al., 2001; Liaw et al., 1998; Likui et al., 2011; Midwood et al., 2004; Zhang et al., 2014; Zhivkova‐Galunska et al., 2010), persistent OPN production by tubular epithelial cells could have detrimental effects in PKD by promoting cyst epithelial cell proliferation, cyst growth, or fibrosis. To test this hypothesis, we conducted both in vitro and in vivo experiments to better understand how OPN contributes to epithelial cell proliferation and cystic kidney disease progression in mice.

## 2 METHODS

### 2.1 Animal preparation and study protocol

All mice were maintained in accordance with recommendations in the “Guide for Care and Use of Laboratory Animals,” from the Institute on Laboratory Animal Resources, National Research Council (National Academy Press, 1996), and all animal protocols were reviewed and approved by the University of Kansas Medical Center (KUMC) Institutional Animal Care and Use Committee prior to the commencement of this research.

*Pcy/pcy* mice possess a deletion of the nephrocystin‐3 (*Nphp3*) and develop kidney cysts that primarily originate in the collecting ducts. These mice are maintained on a CD1 background and display a slowly progressive and consistent PKD phenotype (Raman et al., 2017; Wallace, Hou, et al., 2008). Mice with *Spp1* gene deletion (C57B/6 J background) were originally obtained from Jackson Labs and bred to CD1 wild‐type mice for 6 generations prior to breeding to the *pcy/pcy* strain to generate study mice. The *RC/RC* strain (used to validate kidney OPN expression patterns in PKD) is a murine model exhibiting a clinically relevant mutation in the *Pkd1* gene that drives the production of a mutant polycystin‐1 protein and results in the development of slowly‐progressive PKD (Hopp et al., 2012). Study mice were fed a standard chow diet (Teklad Rodent Diet 8604) beginning at weaning and maintained on a 12‐h light/dark cycle according to the KUMC lab animal facility protocol. All experiments included both male and female mice. Mice were euthanized by exsanguination and blood and tissues were collected at 20 or 40 weeks‐of‐age.

### 2.2 Tissue processing and histology

Kidneys were fixed in 4% paraformaldehyde for 24 h, embedded in paraffin, and cut into 5‐μm sections. H&E and Von Kossa staining of tissue sections were performed using standard published protocols. For quantification of cyst burden, tissue sections were stained with hematoxylin and eosin, and images were collected using a dissecting microscope connected to a digital camera (Leica Microsystems, Buffalo Grove, IL). Total number of cysts and cystic cross‐sectional surface area (SA) per kidney section were determined by an observer, blinded to the identity of the slides, using a morphometric analysis system (AnalySis, Soft Imaging System, Lakewood, CO).

For OPN immunohistochemistry (IHC), sections were de‐paraffinized and steamed in 0.01 M citrate buffer (pH = 6.0) for 20 min then incubated in 3% H_2_O_2_ for 10 min followed by incubation in horse serum for 1 h at room temperature. Sections were then incubated with anti‐OPN (Catalog# AF808, R&D Systems; Minneapolis, MN) overnight at 4°C. An ImmPRESS® HRP horse anti‐goat IgG secondary antibody (catalog# MP‐7045; Vector Laboratories, Burlingame, CA) was applied for 1 h at room temperature followed by the incubation with DAB substrate and hematoxylin counterstaining. Images for IHC quantification were taken at 20x magnification using a Lumenera INFINITY‐5 camera.

Interstitial fibrosis was assessed by picrosirius red staining with subsequent imaging using NIH ImageJ, version 1.43 (http://rsb.info.nih.gov) and quantified by a renal pathologist (TAF) blinded to specimen identity. The method of fibrosis assessment was adapted from prior recommendations for renal biopsy evaluation of PKD kidneys (Wilson et al., 1992). Briefly, images were imported into ImageJ, mid‐sagittal kidney sections stained with picrosirius red were visually inspected and areas of pathologic fibrosis were outlined. Following identification of fibrotic areas, ImageJ was used to calculate the area of fibrosis as a percentage of the total non‐cystic cross‐sectional area of the kidney.

For assessment of Ki‐67 expression, immunohistochemistry was performed on formalin‐fixed, paraffin‐embedded kidney sections of 5 μm thickness. Sections were deparaffinized with xylene, rehydrated with decreasing ethanol concentration (100%, 95% and 70%) and washed twice in PBS. Antigen retrieval was performed by steaming in 0.01 M citrate buffer (pH 6.0) for 20 min. Sections were quenched by incubation in 3% H_2_0_2_ for 10 min. Sections were then incubated overnight at 4°C with an anti‐Ki‐67 antibody (diluted 1:100 in TBS; Cell Signal, #12202S) and were subsequently washed and incubated with ImmPRESS horseradish peroxidase horse anti‐rabbit IgG secondary antibody (diluted 1:3 in TBS; Vector Laboratories, #MP‐7451) for 1 h at room temperature. Sections were finally developed with DAB substrate and hematoxylin counterstaining. Images for quantification were taken at 10x magnification using a Leica Flexcam C5 camera. Five images were taken at predetermined locations for each sample to include cortical and medullary portions of kidney tissue. Positively stained cyst‐lining epithelial cells were counted using ImageJ software. Positive cells per image were averaged for the five images of each kidney and expressed as a single value for each kidney.

Immunohistochemistry for CD68 expression was performed on formalin‐fixed, paraffin‐embedded kidney sections of 5 μm thickness. Sections were deparaffinized with xylene, rehydrated with decreasing ethanol concentration (100, 95 and 70%) and washed twice in PBS. Antigen retrieval was performed by steaming in 0.01 M citrate buffer (pH 6.0) for 20 min. Sections were quenched by incubation in 3% H_2_0_2_ for 10 min. Sections were then incubated overnight at 4°C with an anti‐CD68 antibody (1:500 dilution; Abcam, #ab125212) and were subsequently washed and incubated with ImmPRESS horseradish peroxidase horse anti‐rabbit IgG secondary antibody (undiluted; Vector Laboratories, #MP‐7801) for 1 h at room temperature. Sections were finally developed with DAB substrate and hematoxylin counterstaining. Images for quantification were taken at 5x magnification using a Leica Flexcam C5 camera. Four images were taken at predetermined locations for each sample to include cortical and medullary portions of kidney tissue. Positively stained cells were counted using ImageJ software. Positive cells per image were averaged for the 4 images of each kidney and expressed as a single value for each kidney.

TUNEL staining was performed on formalin‐fixed, paraffin‐embedded kidney sections according to the manufacturer's protocol (In Situ Cell Death Detection Kit, Fluorescein; Roche #11684795910). Treatment with DNase‐I was used as a positive control to ensure proper detection of apoptotic cells. Images were taken from four pre‐determined areas of each kidney and positively stained cyst epithelial cells were counted from a total of 40 cysts in each kidney. The average number of positive cyst‐epithelial cells was calculated for the four images and expressed as a single value for each kidney.

### 2.3 Serum biochemistries

BUN, creatinine, calcium, and phosphate were measured using an Integra 400 Plus Bioanalyzer (Roche Diagnostics, Indianapolis, IN) by the UT Southwestern Metabolic Phenotyping Core. Intact FGF23 was measured by ELISA (QuidelOrtho, #60–6800) and PTH by the Mouse PTH 1–84 ELISA Kit (QuidelOrtho, #60–2305).

### 2.4 Micro‐CT imaging

Whole formalin‐fixed kidneys were individually wrapped and heat‐sealed in cling film to prevent dehydration and stacked in a sample container for batch analysis by a Scanco micro‐CT 40 (Scanco Medical, Brüttisellen, Switzerland). A batch control file for kidney samples with the following specifications was used: energy intensity 45 kVp, 88 μA and 4 W; FOV/Diameter of 12 mm; voxel (VOX) resolution size is 6 μM; integration time of 300 mS. Bone samples (femur diaphysis) batch control parameters were 55 kVp, 72 μA and 4 W; FOV/Diameter of 12 mm; voxel (VOX) resolution size is 6 μM; integration time of 300 mS. Following raw data acquisition and computer reconstruction the output files were contoured and defined using the Scanco software morph and integration functions. For kidney 3D image and quantitative analyses, a script file with the following specifications was used: gauss sigma = 0.8, gauss support = 1; lower threshold = 444 mg HA/ccm, upper threshold 1000 mg HA/ccm. For bone 3D image and quantitative analyses, the following script file specifications were used: gauss sigma = 0.8, gauss support = 1; lower threshold = 280 mg HA/ccm, upper threshold 1000 mg HA/ccm.

### 2.5 Cell culture experiments

Primary cultures of ADPKD and normal human kidney (NHK) cells were generated by PKD Biomarkers and Biomaterials Core in the Kansas PKD Research and Translational Core Center at the Kansas University Medical Center (KUMC), as described previously (Reif et al., 2011; Wallace & Reif, 2019). The use of de‐identified clinical specimens for research complies with federal regulations and was determined to be “not human subjects research” by regulatory agencies and the Institutional Review Board at KUMC. For cell culture experiments, 0.25 mL of normal or cystic kidney epithelial cells were plated at a density of 1 × 10^6^ cells/mL in DMEM/F12 + 1% FBS + P/S in each well of a 24‐well plate and cells were left undisturbed for 24 h to facilitate attachment. Next, the original culture medium was aspirated and replaced with fresh medium containing a lower serum concentration (0.05% FBS) for an additional 24 h. Cells were then treated with different concentrations of recombinant human OPN (SRP3131; Sigma‐Aldrich) for 24 h. To dissociate cells from the plate, 0.15 mL trypsin–EDTA was added per well and incubated at 37°C for 30 min or until the cells formed a single‐cell suspension. Quantification of total cells per well was accomplished using an automated cell counter (Bio‐Rad TC20).

### 2.6 Quantitative real‐time PCR

Kidney specimens for gene expression analysis were snap frozen in liquid nitrogen and stored at −80°C until further processing. Total RNA was extracted following homogenization using TRI‐Reagent (Molecular Research Center, Cincinnati, OH) and treated with RNase‐free DNase (Qiagen, Valencia, CA). First strand cDNA was synthesized using iScript cDNA Synthesis Kit (Bio‐Rad, Hercules, CA), with 1 μg of RNA used for each reverse‐transcriptase reaction. PCR reactions contained 100 ng cDNA, 300 nM of each primer, and 1X iQ™ SYBR® Green Supermix (Bio‐Rad) in 50 μL. The threshold cycle (Ct) of each gene product was normalized to the Ct for hypoxanthine guanine phosphoribosyl transferase (*HPRT*) for all qRT‐PCR experiments. Gene primer sequences are: *HPRT* forward: 5′‐TGATAGATCCATTCCTATGACTGTAGA‐3′, *HPRT* reverse: 5′‐AAGACATTCTTTCCAGTTAAAGTTGAG‐3′; *Col1α1* forward: 5′‐CATGTTCAGCTTTGTGGACCT‐3′, *Col1α1* reverse: 5′‐GCAGCTGACTTCAGGGATGT‐3′; *TGF‐β* forward: TGGAGCAACATGTGGAACTC‐3′, *TGF‐β* reverse: GTCAGCAGCCGGTTACCA; *Acta2 forward*: 5′‐CTCTCTTCCAGCCATCTTTCAT‐3′, *Acta2* reverse: 5′‐TATAGGTGGTTTCGTGGATGC‐3′; *Adgre1 forward*:5′‐CTTTGGCTATGGGCTTCCAGTC‐3′, *Adgre1 reverse* 5′‐GCAAGGAGGACAGAGTTTATCGTG‐3′.

### 2.7 Statistical analysis

Differences between multiple groups were evaluated by one‐way ANOVA with Dunnett's multiple comparison test. Differences between two groups were evaluated by two‐sided Student's *t*‐test (for data with a Gaussian distribution) or Mann–Whitney test (for data with a non‐Gaussian distribution). Computations were performed using Prism 9 software (GraphPad Software, San Diego, CA) and presented as mean ± SD unless otherwise specified.

## 3 RESULTS

### 3.1 Osteopontin expression is increased in tubular epithelial cells of mice with cystic kidney disease

**FIGURE 1:**
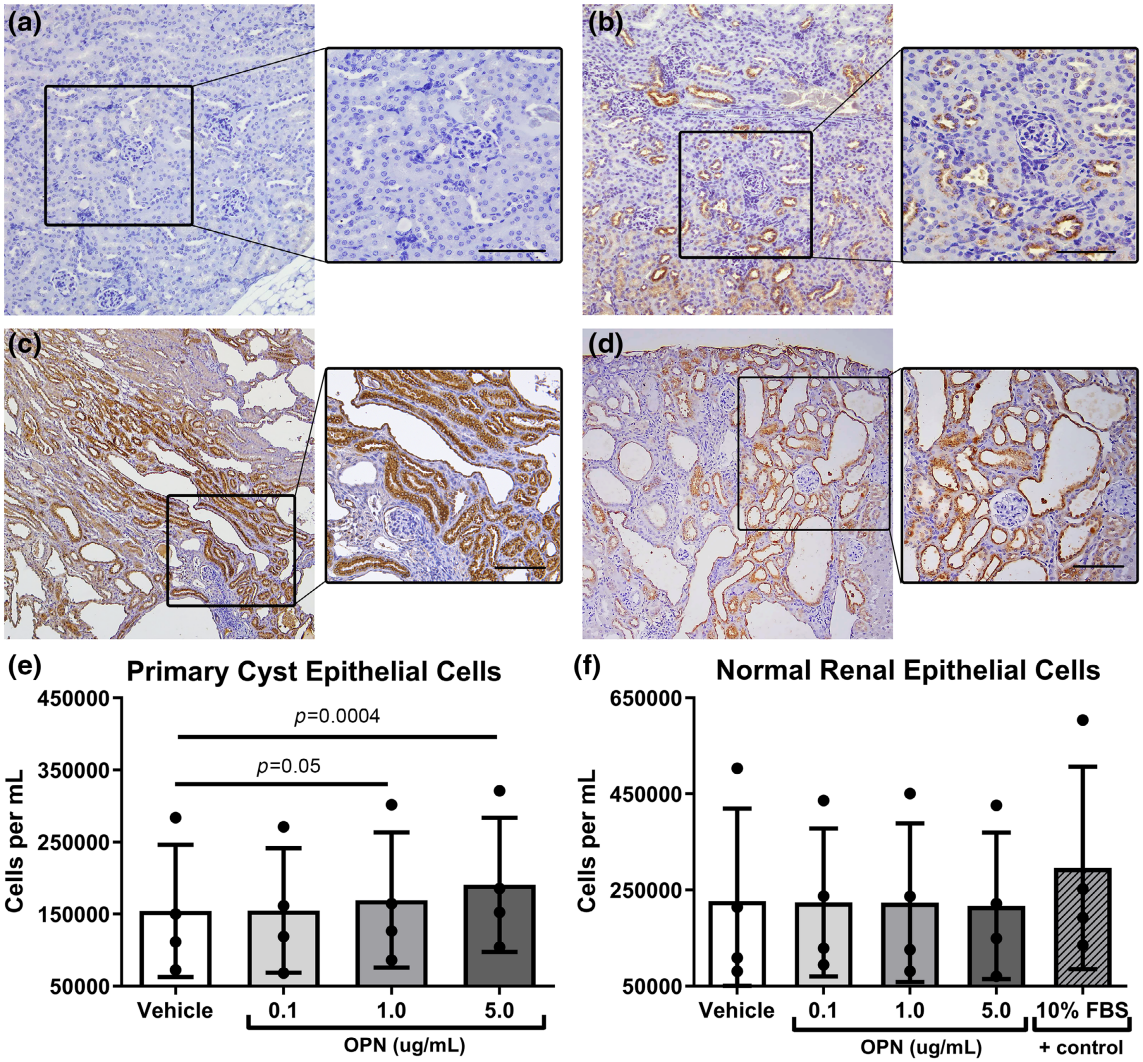
Osteopontin (OPN) expression is increased in kidneys from mice with cystic kidney disease and stimulates cyst epithelial cell proliferation in vitro. Immunohistochemistry (IHC) of OPN protein expression (brown) in kidneys from (a) mice with *Spp1* (OPN) gene deletion, (b) wild‐type controls, (c) *pcy/pcy* mice (non‐orthologous PKD model), and (d) *RC/RC* mice (orthologous PKD model) (enlarged inset is 20× magnification; scale bar = 100 μm). Further in vitro experiments were performed to test the effect of increasing concentrations of OPN on cellular proliferation in (e) human primary cyst epithelial cells and (f) primary human tubular epithelial cells from normal kidneys (analyzed by 1‐way paired ANOVA).

We performed immunohistochemistry staining of paraffin‐embedded kidney sections from two separate mouse models of cystic kidney disease (*pcy/pcy* and *RC/RC* mice), along with mice with global OPN deletion (*Spp1* ^*−/−*^ mice; negative control) and wild‐type controls to determine how the localization of OPN expression in the kidney is altered in cystic kidney disease (Figure 1a–d). As anticipated, we observed no OPN staining in kidneys from *Spp1* ^*−/−*^ mice (Figure 1a). Moreover, as previously described (Xie et al., 2001), we found OPN expression to be restricted to distal tubular epithelial cells in wild‐type mice (Figure 1b). By contrast, *pcy/pcy* and *RC/RC* kidneys exhibited intense OPN staining in epithelial cells located in all tubular segments, including cyst‐lining epithelial cells (Figure 1c,d).

### 3.2 Osteopontin stimulates the proliferation of human PKD cyst epithelial cells

To determine the direct effect of OPN on PKD cell proliferation, we conducted in vitro experiments testing the various concentrations of OPN on human autosomal dominant PKD primary cyst epithelial cells or normal renal epithelial cells. We observed OPN to produce a dose‐dependent increase in the proliferation of the PKD cells. By contrast OPN did not affect the proliferation of the normal renal epithelial cells (Figure 1e,f).

### 3.3 Gene knockout of osteopontin reduces kidney cyst burden in *pcy/pcy* mice

**FIGURE 2:**
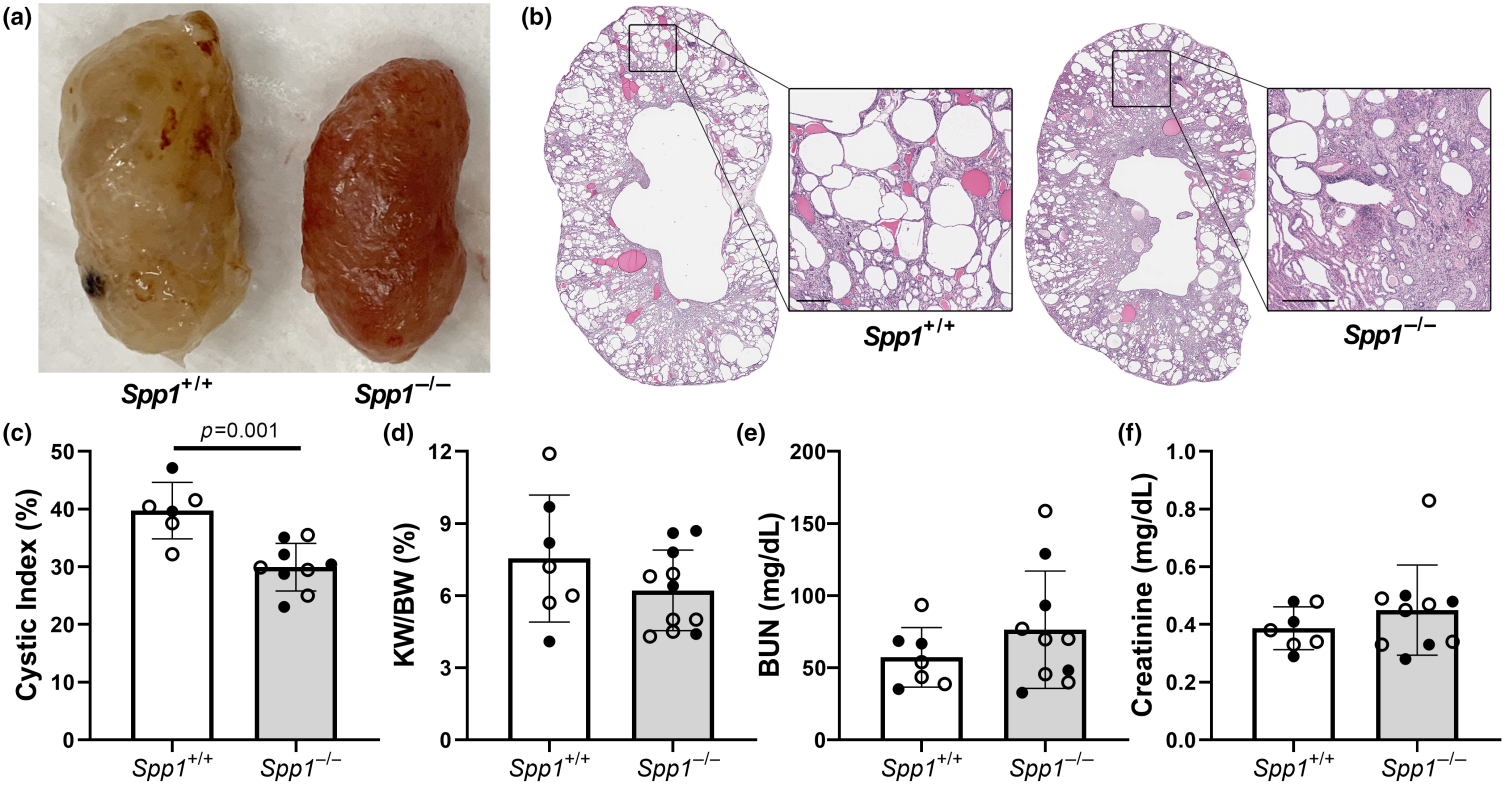
Kidney cyst burden is reduced in *pcy/pcy* mice exhibiting OPN (*Spp1*) deletion. (a) Gross tissue evaluation of kidneys from *pcy/pcy* mice with and without *Spp1* expression. (b, c) H&E staining of kidney sections to assess cystic index in study mice (magnified images taken at 10x; error bar = 500 μm). Additional kidney related outcomes were conducted, including (d) two‐kidney to total body weight ratio (KW/BW), (e) BUN, and (f) serum creatinine (analyzed by Student's *t*‐test; closed circles = males, open circles = females).

We mated OPN knockout (*Spp1* ^*−/−*^) mice to *pcy/pcy* mice to determine if the loss of OPN affected the progression or fibrosis of PKD. At 40 weeks of age, kidneys of *pcy/pcy; Spp1* ^*−/−*^ mice appeared smaller and reddish brown in color compared to *pcy/pcy; Spp1* ^*+/+*^, which appeared pale and irregular due to larger surface cysts (Figure 2a). Kidney histology revealed a reduced cyst burden in *pcy/pcy; Spp1* ^*−/−*^ compared to *pcy/pcy; Spp1* ^*+/+*^ mice (Figure 2b,c). However, we found no statistically significant difference in two kidney‐to‐total body weight (KW/BW) of *pcy/pcy* mice with *Spp1* deletion (Figure 2d); and there was no statistical difference in BUN and serum creatinine values between these two groups (Figure 2e,f).

### 3.4 Kidney fibrosis is exacerbated by OPN deletion in *pcy/pcy* mice

**FIGURE 3:**
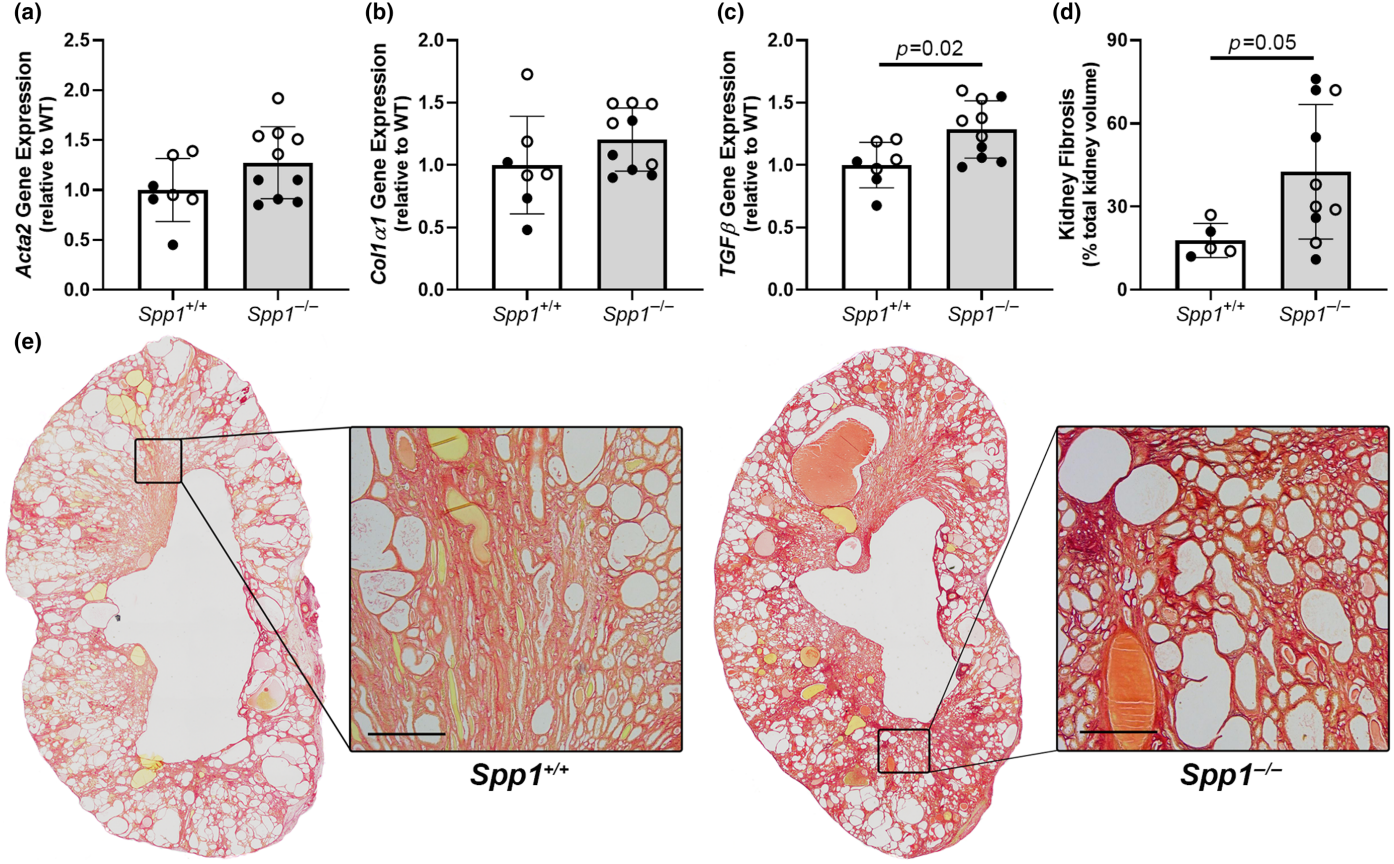
Kidney fibrosis is increased in *pcy/pcy* mice in the absence of OPN. Quantitative RT‐PCR analysis to evaluate gene expression for key fibrosis mediators demonstrated increased kidney expression of genes encoding (a) α‐smooth muscle Actin (*Acta2*), (b) α1 subunit of type I collagen (*Col1α1*), and (c) transforming growth factor β (*TGFβ*). (d, e) Quantification of fibrosis by picrosirius red staining of kidney histology sections from *pcy/pcy* mice with and without OPN (*Spp1*) expression (analyzed by Student's *t*‐test; closed circles = males, open circles = females).

Despite a reduction in cyst burden, *pcy/pcy; Spp1* ^*−/−*^ kidneys exhibited increased expression of genes involved in kidney fibrosis, including α‐smooth muscle actin (*Acta2*), collagen 1 α1‐subunit (*Col1α1*), and transforming growth factor‐β (*TGFβ*) (Figure 3a–c). Quantification of kidney fibrosis by picrosirius red staining confirmed increased fibrosis in *pcy/pcy; Spp1* ^*−/−*^ mice compared to *pcy/pcy; Spp1* ^*+/+*^ mice (Figure 3d,e).

### 3.5 Effect of OPN deletion on mineral metabolism and tissue mineralization in *pcy/pcy* mice

**FIGURE 4:**
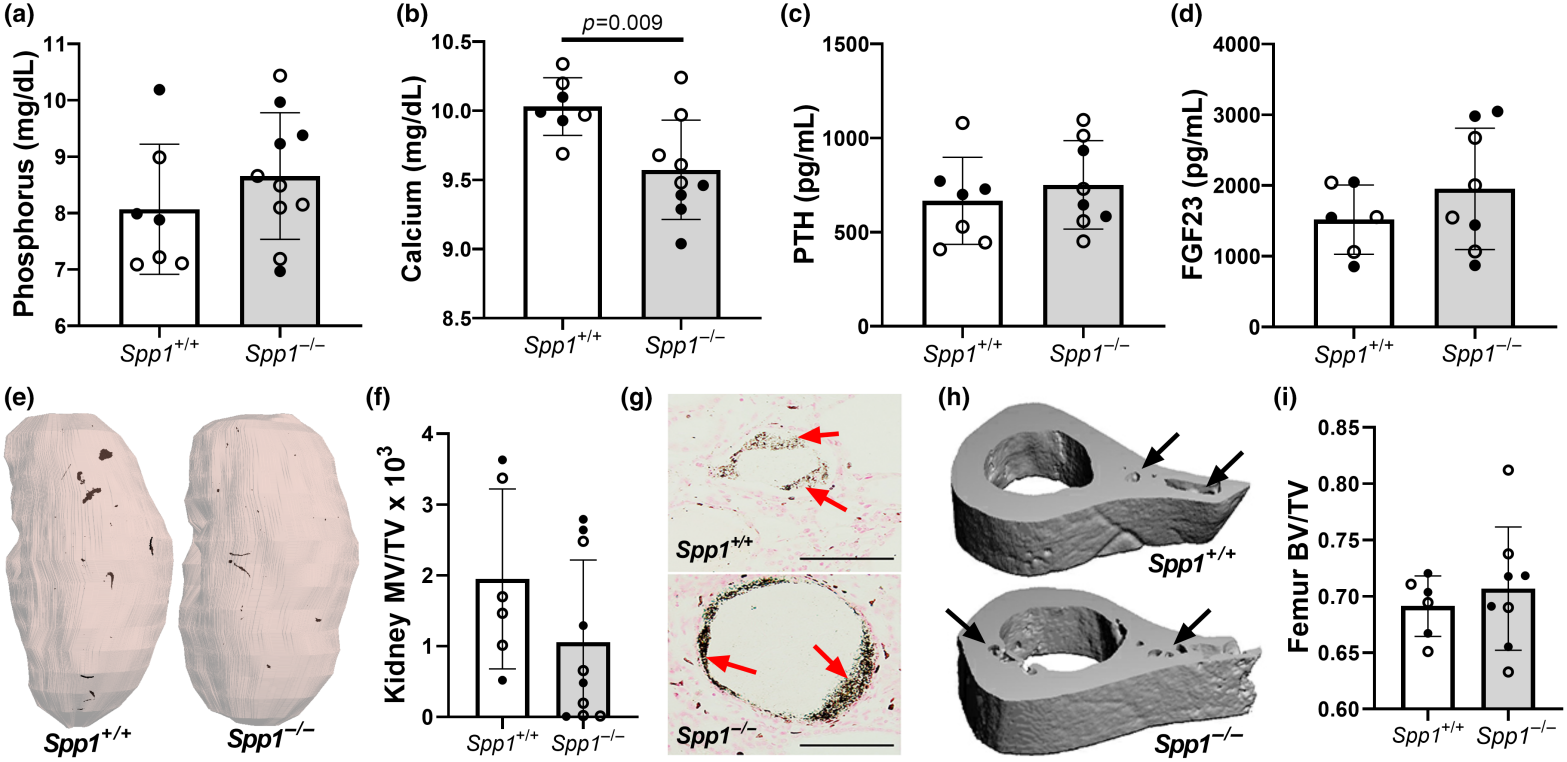
Osteopontin deficiency has minimal effect on mineral metabolism parameters in *pcy/pcy* mice. Serum measurements of mineral metabolism parameters were conducted, including (a) phosphorus, (b) calcium, (c) parathyroid hormone (PTH), and (d) intact fibroblast growth factor 23 (FGF23). (e, f) Evaluation of kidney mineral deposition revealed only sparse kidney mineral deposits in both groups. (g) Von Kossa staining of kidney sections showing mineral deposits (red arrows) attached to epithelial surfaces within cysts. (h, i) Evaluation of bone mineral content in femurs from study mice showed evidence of cortical bone porosity (black arrows) in both groups (analyzed by Student's *t*‐test; closed circles = males, open circles = females).

We assessed the effect of OPN deletion on mineral metabolism parameters in *pcy/pcy; Spp1* ^*−/−*^ mice and *pcy/pcy; Spp1* ^*+/+*^ mice at 40 weeks of age, including measurements of serum phosphorus, calcium, parathyroid hormone (PTH), and fibroblast growth factor 23 (FGF23) (Figure 4a–d). There was a modest, but significant, reduction in serum calcium in the *pcy/pcy* mice with the loss of OPN (Figure 4b), whereas other measured biochemical parameters were unchanged between the study groups. Micro‐CT evaluation of whole kidneys revealed high variability in the extent of mineral deposition among study mice with no discernable difference in kidney mineralization between the two groups of interest (Figure 4e,f). Von Kossa staining of kidney tissue sections demonstrated that mineral aggregates were primarily attached to epithelial surfaces within cyst lumens (Figure 4g). There was no effect of OPN knockout on the bone volume of femurs from *pcy/pcy* mice with and without OPN expression indicating that the loss of OPN did not impact overall bone mineralization (Figure 4h,i).

### 3.6 Kidney macrophage numbers are unaffected by OPN deletion in *pcy/pcy* mice

**FIGURE 5:**
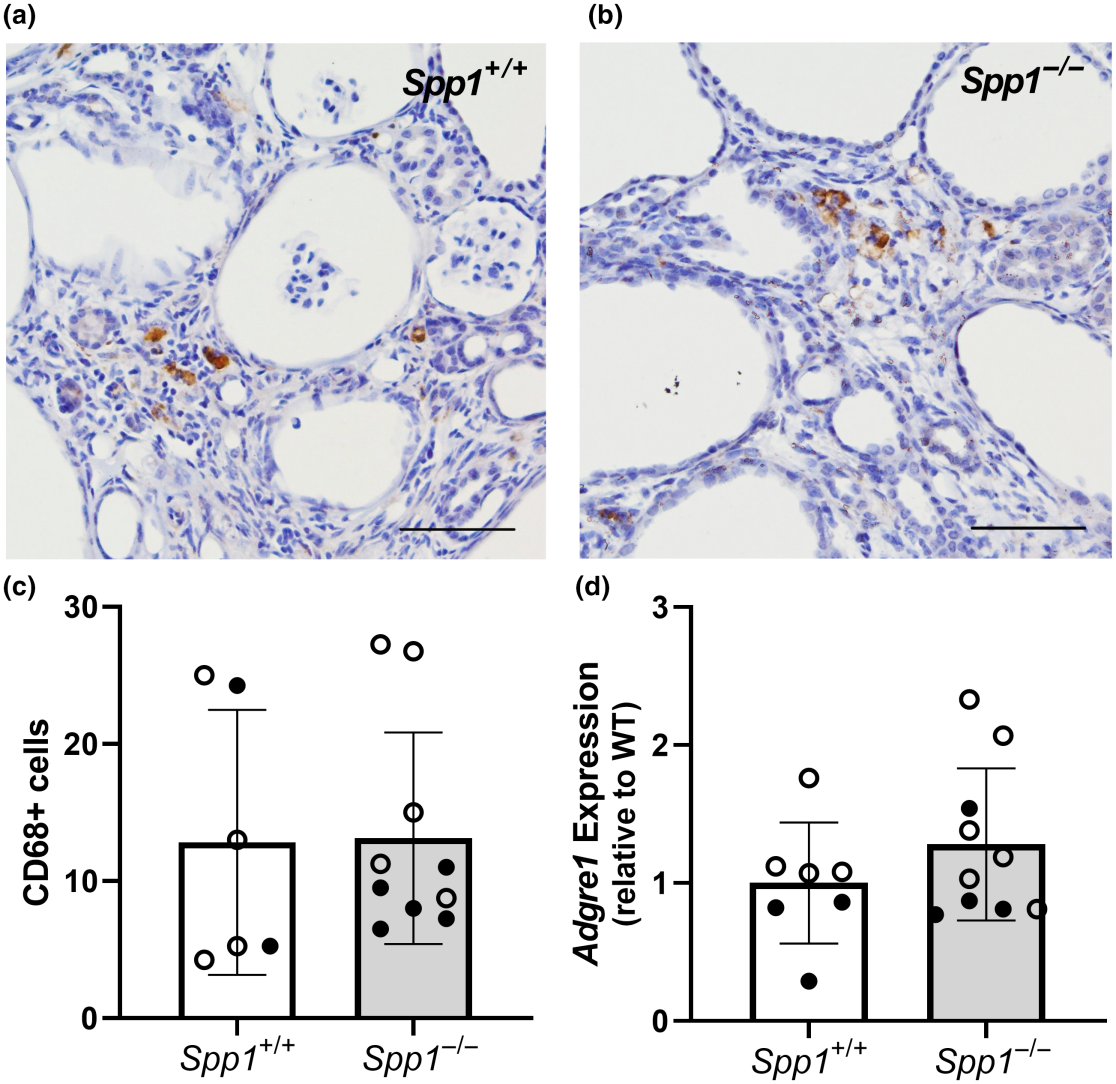
Osteopontin deficiency does not alter kidney macrophage numbers in *pcy/pcy* mice. Immunohistochemistry staining of kidney sections for macrophage marker CD68 in *pcy/pcy* mice (a) with and (b) without OPN expression (scale bar = 100 um), along with (c) subsequent quantification of CD68‐positive cells from these sections (data expressed as positive cells per kidney section). (d) Assessment of kidney gene expression by qRT‐PCR for *Adgre1* (F4/80), an alternative macrophage marker (closed circles = males, open circles = females).

To determine if increased kidney fibrosis in *pcy/pcy* mice with OPN deletion was a result of alterations to macrophage numbers or localization within the kidney, we performed macrophage phenotyping by assessing *Adgre1* (F4/80) gene expression by qRT‐PCR and evaluating CD68 expression by immunohistochemistry (Figure 5). We observed no obvious difference in total kidney macrophage numbers as assessed by CD68 staining (Figure 5a–c) or *Adgre1* gene quantification (Figure 5d).

### 3.7 Kidney fibrosis appears at an early age in *pcy/pcy* mice with OPN deletion

**FIGURE 6:**
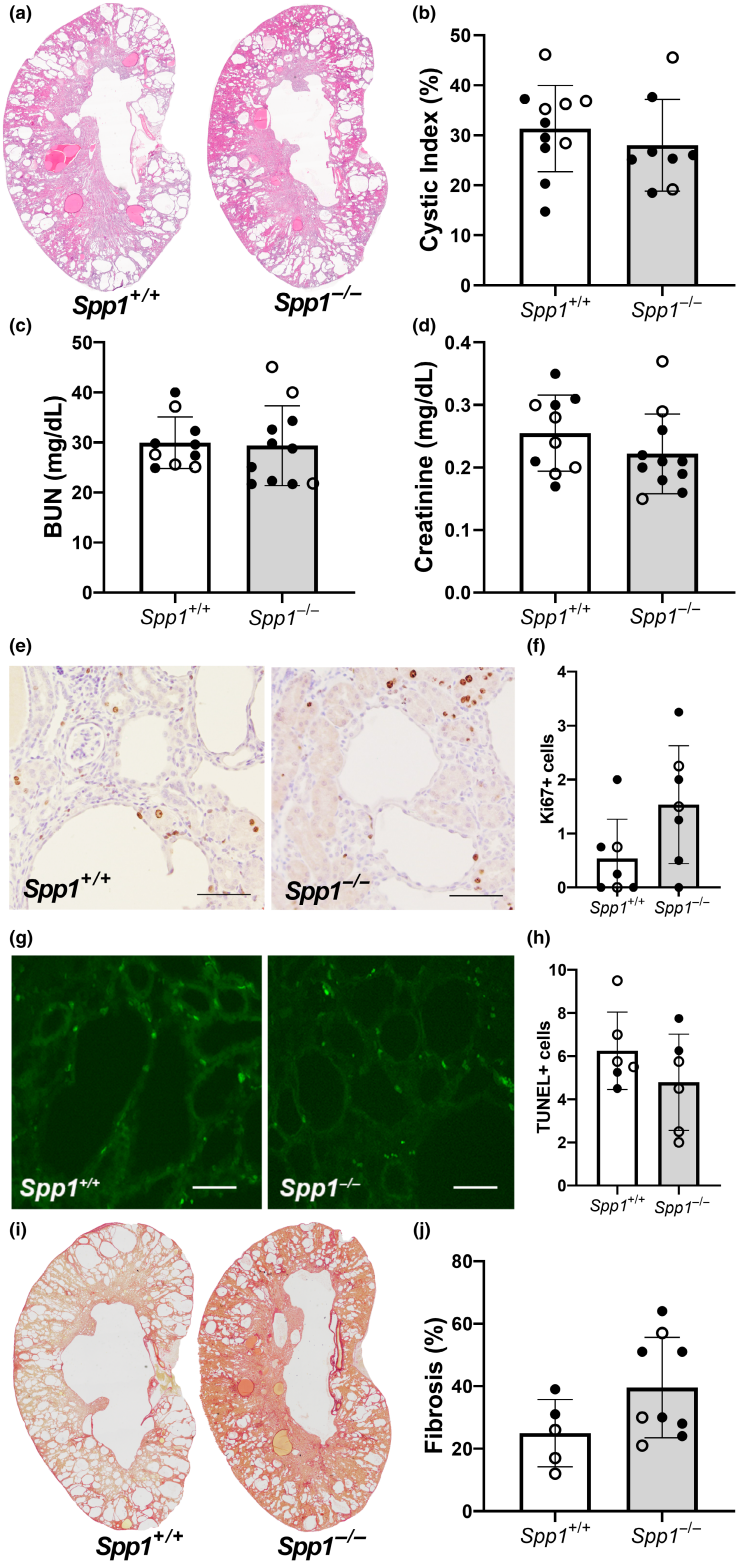
Kidney fibrosis is an early phenotypic finding in *pcy/pcy* mice with OPN deletion. Evaluation of kidney disease parameters at an earlier time point (20 weeks‐of‐age) in *pcy/pcy* mice with and without OPN expression, including (a, b) cyst burden by histology, (c, d) quantification of biochemical markers of kidney function, (e, f) Ki‐67 staining to assess proliferation of cyst‐lining epithelial cells, (g, h) TUNEL‐staining to quantify apoptosis of cyst lining cells (data expressed as positive cells per kidney section for both Ki‐67 and TUNEL stains), and (i, j) picrosirius red staining to evaluate kidney fibrosis (analyzed by Student's *t*‐test; closed circles = males, open circles = females).

To further examine the relative timing of cyst formation and fibrosis in *pcy/pcy* mice with and without OPN expression, we assessed kidney phenotypes and markers of kidney function at an earlier timepoint (20 weeks of age). These analyses revealed no definitive difference in kidney cyst burden or kidney function (BUN or serum creatinine) at this age (Figure 6a–d). Moreover, our assessment of tubular epithelial cell proliferation and apoptosis by Ki‐67 and TUNEL staining, respectively, found no definitive difference in these markers between study groups (Figure 6e–h). Despite these between‐group similarities, there was already a pattern of increased kidney fibrosis in 20‐week‐old *pcy/pcy* mice with OPN deletion (Figure 6i,j).

## 4 DISCUSSION

Osteopontin is a widely expressed, multifunctional protein that is dramatically upregulated by tubular epithelial cells in the setting of kidney injury, including in PKD (Cowley Jr. et al., 2001; Stubbs et al., 2022). Cancer biology researchers have long been interested in OPN given its observed functions to accelerate cellular division, inhibit apoptosis, and drive tumor propagation (Cui et al., 2007; Denhardt et al., 2001; Liaw et al., 1998; Likui et al., 2011; Midwood et al., 2004; Shevde & Samant, 2014; Zhang et al., 2014; Zhivkova‐Galunska et al., 2010). Moreover, OPN has received substantial attention from immunology researchers given its role in coordinating macrophage trafficking and associated innate immune responses (Denhardt et al., 2001; Liaw et al., 1998; Okada et al., 2000; Scatena et al., 2007). Importantly, aberrant cellular division, dysregulated repair, and macrophage signaling are prominent pathologic features contributing to kidney disease progression in PKD.

In this investigation, we confirmed increased OPN expression in the cystic kidneys of *pcy/pcy* mice. Consistent with prior reports, OPN expression was localized to distal tubule segments in wild‐type mice (Xie et al., 2001); however, both *pcy/pcy* and *RC/RC* mice with cystic kidney disease exhibited widespread OPN expression in all tubular segments (Figure 1b–d). In cell culture studies examining the potential contribution of this OPN expression to epithelial cell division, we found a stepwise increase in epithelial cell numbers with increasing concentrations of OPN in cyst‐derived epithelial cells, but no similar response in tubular cells harvested from control kidneys (Figure 1e,f). While the exact mechanism for this varied response remains unclear, we can speculate a potential mechanism based on existing knowledge. OPN contains an RGD domain that binds α_V_‐integrins, which are key mediators of mitogenic signals (such as GSK3β and mTOR) and macrophage recruitment that contribute to cyst growth. We have previously observed human ADPKD cells to exhibit a 9‐fold higher expression of α_V_‐integrin compared to normal human kidney cells (Wallace, Quante, et al., 2008); thus, we suspect that enhanced integrin signaling may contribute to the unique response of human ADPKD cells to OPN. In further support of this hypothesis, we previously demonstrated that stimulating mitogenic pathways in an α_V_‐integrin‐dependent manner promotes PKD progression in mice (Raman et al., 2017; Wallace, Quante, et al., 2008).

We discovered that *pcy/pcy* mice lacking OPN expression exhibited an approximate 25% reduction in cystic index at 40 weeks‐of‐age (Figure 2c). Interestingly, despite an obvious decrease in cyst burden in *pcy/pcy* lacking *Spp1*, our observation of slightly higher BUN and serum creatinine measurements suggested *worse* kidney function in this group (Figure 2e,f). Similarly, the trend towards higher serum phosphorus (Figure 4a) and lower serum calcium (Figure 4b), would be consistent with a more rapid decline in kidney function in *pcy/pcy* mice with *Spp1* deletion. We suspect that our studies were simply underpowered to detect small differences in most of these biochemical parameters and that these trends are likely explained by the prominent kidney fibrosis phenotype in *Spp1* ^*−/−*^ mice (Figure 3). Of note, kidney fibrosis is a prominent feature of nephronophthisis, which is the form of cystic kidney disease resulting from mutations in the NPHP genes (as present in *pcy/pcy* mice). As such, multiple studies have demonstrated that substantial kidney fibrosis occurs at a much earlier stage in patients with this disorder compared to other forms of cystic kidney disease, such as ADPKD (Slaats et al., 2016). Our evaluation of the kidney phenotype of these animals at the 20‐week timepoint (Figure 6) is consistent with the observations in patients with nephronophthisis. We found kidney fibrosis to be very prominent by 20 weeks in *pcy/pcy* mice lacking OPN expression, even before detectable differences in cyst burden between study groups. One possible explanation for the disconnect between cyst burden and fibrosis in this model could be that the development of early interstitial fibrosis impedes cyst growth, perhaps by stiffening of the extracellular matrix or the promotion of signals that slow epithelial cell proliferation or fluid secretion. Since we did not observe any obvious alteration to the proliferation or apoptosis of epithelial cells or macrophage accumulation in kidneys from *pcy/pcy*; *Spp1* ^*−/−*^ mice compared to pcy/pcy mice with intact *Spp1* expression (Figures 5 and 6), the etiology of the early kidney fibrosis in this model remains uncertain and will require more extensive investigation.

In a prior study, we found that OPN serves a crucial role in the prevention of kidney mineral deposition in chronic kidney disease (Stubbs et al., 2022). Based on this previous observation, along with the known effect of tubular crystal formation to trigger innate immune responses in the kidney (Anders et al., 2018; Mulay et al., 2013, 2014), we initially hypothesized that enhanced kidney fibrosis in *pcy/pcy* mice with OPN deletion could result from aberrant kidney mineral deposition in this group. However, our evaluation of kidney mineral content by both high‐resolution μCT (Figure 4e,f) and Von Kossa tissue staining (Figure 4g) demonstrated no apparent difference in kidney mineral content in *pcy/pcy* mice with and without *Spp1* deletion. We speculate that the lack of significant kidney mineral deposition in *Spp1* ^*−/−*^ mice with cystic kidney disease was the result of substantially preserved kidney function in this model. Prior studies have demonstrated that tubular mineral content reaches supersaturation as eGFR is reduced and the functional nephron mass reaches a critical threshold (Bank N et al., 1978). It is also plausible that kidney mineral deposition was in fact enhanced with OPN deletion, but that resulting mineral aggregates were below the lower limit of detection by μCT or histology analyses. We speculate that evaluating this model at an older age or in the setting of high dietary phosphate consumption could elicit a significant nephrocalcinosis phenotype in the *pcy/pcy; Spp1* ^*−/−*^ mice.

Binding of OPN to α‐integrins is a potent chemotactic stimulus for macrophage recruitment and propagates tissue fibrosis (Giachelli et al., 1998; Lancha et al., 2014; Liaw et al., 1995, 1998; Lopez et al., 2013; Lund et al., 2013; Scatena et al., 2007; Urtasun et al., 2012; Wang et al., 2014; Zhang et al., 2014). Accordingly, anti‐OPN therapies attenuate inflammation and fibrosis in kidney injury models (Okada et al., 2000; Yu et al., 1998). Given this prior literature suggesting OPN is involved in the coordination of innate immune responses, along with the known contribution of macrophages to PKD progression (Karihaloo et al., 2011; Swenson‐Fields et al., 2013; Yang et al., 2018), we performed additional experiments to test the effect of OPN deletion on kidney macrophage numbers and localization within the kidney. These analyses revealed no obvious difference in total macrophage counts by immunohistochemistry or qRT‐PCR gene expression for key macrophage markers (CD68 and F4/80, respectively) (Figure 5). Moreover, our evaluation of macrophage localization within the kidneys from these groups demonstrated the presence of macrophages in both the interstitial space and cyst lumen; however, we appreciated no difference in the abundance of macrophages in these separate compartments. It remains plausible that OPN deletion resulted in some phenotypic change in existing macrophages that could not be appreciated by cell quantification alone, so further studies will be required to more thoroughly examine the impact of changes in OPN expression on local macrophage function in cystic kidneys.

The current investigation has both important strengths and limitations. Strengths of the study include the use of unbiased approaches to assess kidney cyst burden and fibrosis, detailed assessments of kidney phenotypes at two separate timepoints, exploration of multiple potential contributors to kidney fibrosis and cyst development (macrophage recruitment, mineral deposition, epithelial cell proliferation and apoptosis), and a study design that allows for the exclusion of potential confounders that are commonly encountered in human studies (i.e., dietary influences, genetic variability, and the presence of disease comorbidities). Limitations of this work include use of a single model of cystic kidney disease, global (non‐targeted) deletion of the *Spp1* gene, no investigation of phenotypic changes in kidney macrophage subpopulations, and lack of a definitive explanation as to how OPN deletion could reduce cyst burden while exacerbating kidney fibrosis in this model.

In summary, our observations in *pcy/pcy* mice with global OPN deletion highlight the potential importance of OPN in contributing to key kidney phenotypes that drive cystic kidney disease progression. Future investigations should focus on validating the role of OPN in other models of cystic kidney disease and identifying how a targeted deletion of OPN in tubular epithelial cells alters our observed outcomes. Further work is needed to clarify the mechanisms responsible for the interplay between local OPN expression, cyst growth, and interstitial fibrosis.

## AUTHOR CONTRIBUTIONS

K.P.J., D.P.W., P.S.R., and J.R.S. conceived and designed research; K.P.J., S.Z., T.S., J.K., and Y.Z. conducted experiments; K.P.J., S.Z., T.S., J.K., Y.Z., T.A.F., K.A.Z., D.P.W., P.S.R., and J.R.S. analyzed data; K.P.J., K.A.Z., D.P.W., P.S.R., and J.R.S. prepared and edited manuscript; J.R.S. prepared figures; all authors reviewed and approved the final manuscript.

## FUNDING INFORMATION

This work was supported by funds from National Institutes of Health (NIH) NIGMS and NIDDK grants (R01DK122212 to JRS; R01DK129255 and K01DK119375‐01A1 to KAZ).

## CONFLICT OF INTEREST STATEMENT

DPW has received research funding from Synkine Therapeutics. All other authors have nothing to disclose.

## Data Availability

Original data in support of the published findings of this study are available from the corresponding author upon reasonable request.
